# Electronic Structure of the Kitaev Material *α*-RuCl_3_ Probed by Photoemission and Inverse Photoemission Spectroscopies

**DOI:** 10.1038/srep39544

**Published:** 2016-12-21

**Authors:** Soobin Sinn, Choong Hyun Kim, Beom Hyun Kim, Kyung Dong Lee, Choong Jae Won, Ji Seop Oh, Moonsup Han, Young Jun Chang, Namjung Hur, Hitoshi Sato, Byeong-Gyu Park, Changyoung Kim, Hyeong-Do Kim, Tae Won Noh

**Affiliations:** 1 Center for Correlated Electron Systems, Institute for Basic Science (IBS), Seoul 08826, Republic of Korea; 2 Department of Physics and Astronomy, Seoul National University (SNU), Seoul 08826, Republic of Korea; 3 Computational Condensed Matter Physics Laboratory, RIKEN, Wako, Saitama 351-0198, Japan; 4 Interdisciplinary Theoretical Science (iTHES) Research Group, RIKEN, Wako, Saitama 351-0198, Japan; 5 Department of Physics, Inha University, Incheon 22212, Republic of Korea; 6 Department of Physics, University of Seoul, Seoul 02504, Republic of Korea; 7 Hiroshima Synchrotron Radiation Center, Hiroshima University, Kagamiyama 2-313, Higashi-Hiroshima 739-0046, Japan; 8 Pohang Accelerator Laboratory, Pohang University of Science and Technology, Pohang 37673, Republic of Korea

## Abstract

Recently, *α*-RuCl_3_ has attracted much attention as a possible material to realize the honeycomb Kitaev model of a quantum-spin-liquid state. Although the magnetic properties of *α*-RuCl_3_ have been extensively studied, its electronic structure, which is strongly related to its Kitaev physics, is poorly understood. Here, the electronic structure of *α*-RuCl_3_ was investigated by photoemission (PE) and inverse-photoemission (IPE) spectroscopies. The band gap was directly measured from the PE and IPE spectra and was found to be 1.9 eV, much larger than previously estimated values. Local density approximation (LDA) calculations showed that the on-site Coulomb interaction *U* could open the band gap without spin-orbit coupling (SOC). However, the SOC should also be incorporated to reproduce the proper gap size, indicating that the interplay between *U* and SOC plays an essential role. Several features of the PE and IPE spectra could not be explained by the results of LDA calculations. To explain such discrepancies, we performed configuration-interaction calculations for a RuCl_6_^3−^ cluster. The experimental data and calculations demonstrated that the 4*d* compound *α*-RuCl_3_ is a *J*_eff_ = 1/2 Mott insulator rather than a quasimolecular-orbital insulator. Our study also provides important physical parameters required for verifying the proposed Kitaev physics in *α*-RuCl_3_.

The honeycomb Kitaev model has attracted significant attention as a feasible model for a quantum-spin-liquid ground state^1^,^2^,^3^,^4^,^5^. In this model, a strong spin-orbit coupling (SOC) plays a critical role because it provides a bond-direction-dependent exchange interaction that results in spin frustration. Various transition-metal compounds, including Na_2_IrO_3_ and Li_2_IrO_3_, have been investigated for possible realization of the Kitaev model^6^,^7^,^8^. These materials contain 5*d*-transition-metal Ir ions that exhibit a large SOC strength *λ*_SOC_ of ~0.4 eV^9^.

*α*-RuCl_3_ has recently been added to the list of Kitaev candidates despite the comparatively modest SOC in the 4*d* Ru ion (*λ*_SOC_ ~ 0.13 eV)^10^. The honeycomb lattice of the system is almost perfect, with an Ru–Cl–Ru angle of approximately 90°^11^,^12^. This near-perfect lattice makes the system ideal for achieving the Kitaev ground state^1^,^2^,^3^,^4^ despite the relatively weak SOC. Numerous experimental studies involving Raman spectroscopy^13^,^14^ or neutron scattering^10^ have indicated that *α*-RuCl_3_ may be close to the Kitaev spin-liquid ground state. To distinguish the interesting Kitaev quantum physics from other classical spin fluctuations, determining the accurate values of physical parameters related to the Kitaev physics, possibly from electronic structure studies, is important. However, there are still some controversies regarding the electronic structure of *α*-RuCl_3_. The magnitude and nature of the band gap remain controversial. An early Hall-effect study of *α*-RuCl_3_ claimed that the band gap should be approximately 0.3 eV^15^. Optical studies found an optical gap of 0.2 eV^16^ that was later revised to 1.0 eV^17^. Recently, an angle-resolved photoemission spectroscopy (ARPES) study showed that the Fermi level *E*_F_ is located 1.2 eV above the valence band maximum, suggesting that the band gap should be larger than 1.2 eV^18^. There are two possible insulating mechanisms for a spin-orbit-coupled *t*_*2g*_^5^ honeycomb system^19^: A *J*_eff_ = 1/2 Mott insulator^20^ and a quasimolecular-orbital band insulator^21^. Whereas the model presumes the *J*_eff_ = 1/2 Mott state^1^, this state has not been experimentally confirmed for this system. Moreover, the physical parameters characterizing the electronic structure and interactions that constitute a key input into the theoretical descriptions of the unconventional magnetism have not yet been determined.

Here, we describe our experimental and theoretical efforts to understand the electronic structure of *α*-RuCl_3_ using both photoemission (PE) and inverse photoemission (IPE) spectroscopies. We observed a band gap of approximately 1.9 eV, much larger than the previously reported values. Local density approximation (LDA) calculations also reveal that the interplay between SOC and electron correlation plays an important role in determining the insulating ground state of *α*-RuCl_3_. However, some features of the PE and IPE spectra could not be fully explained by the band calculations, implying a strongly correlated ground state. To explain such detailed features, we performed configuration-interaction (CI) calculations for a RuCl_6_^3−^ cluster and determined the microscopic parameters relevant to Kitaev physics.

## Results and Discussion

**Figure 1:**
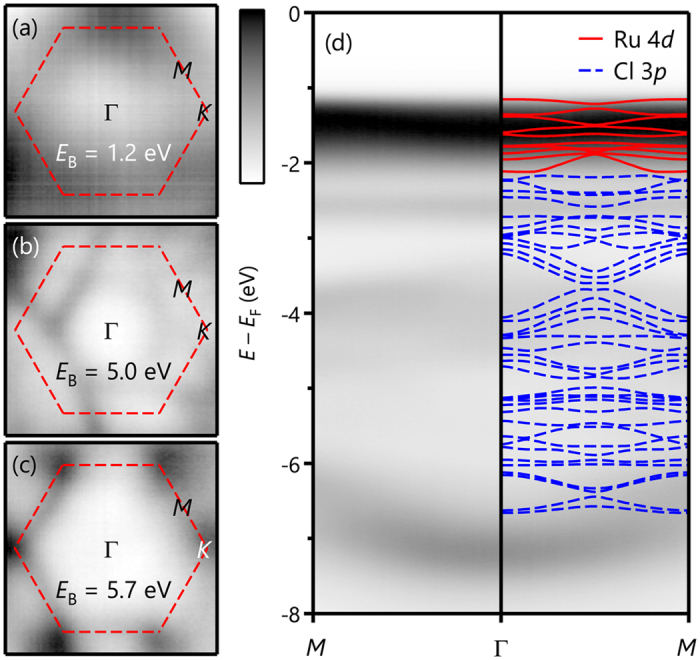
Momentum-dependent electronic structure of *α*-RuCl_3_. ARPES constant-energy maps at different binding energies of (**a**) 1.2 eV, (**b**) 5.0 eV, and (**c**) 5.7 eV. Red-dashed hexagons indicates the Brillouin zone of *α*-RuCl_3_. (**d**) Band dispersions from ARPES along the *M*Γ*M* line. Calculated bands by LDA + SOC + *U (U* *−* *J*_H_ = 4.5 eV) are depicted on the right-hand side of (**d**). Red solid lines and blue dashed lines represent Ru 4*d* and Cl 3*p* bands, respectively. Note that the existence of a flat band at −2.5 eV and the clear separation between Ru 4*d* bands and Cl 3*p* bands are not reproduced in the LDA + SOC + *U* calculations.

The underlying honeycomb symmetry of *α*-RuCl_3_ can be manifested in the constant-energy maps of the ARPES data. Figures 1(a)-(c) show constant-energy maps at the binding energies of *E*_B_ = 1.2, 5.0, and 5.7 eV, respectively. At *E*_B_ = 1.2 eV, the crystal symmetry is not clearly resolved, probably because of the negligible dispersions of the Ru *t*_*2g*_ bands. At the higher binding energies, the maps show a six-fold symmetry originating from the dispersive Cl 3*p* bands. These constant-energy maps confirm the high quality of our sample surfaces investigated.

ARPES spectra along the *M*Γ*M* line in Fig. 1(d) show the nearly flat Ru 4*d* bands near the *E*_F_ and the dispersive Cl 3*p* bands. Most of the Ru 4*d* bands are located between −1.0 and −3.0 eV. In an enlarged view of the Ru 4*d* bands near −1.5 eV, their dispersions are estimated to be approximately 0.1 eV or smaller. By contrast, the Cl 3*p* bands are located between −3.5 and −7.5 eV and are well separated from the Ru 4*d* bands. Compared to the Ru 4*d* bands, the Cl 3*p* bands are highly dispersive. Overall, our ARPES spectra are consistent with recently reported ARPES results^18^,^22^. Note that the energy differences between the five Ru *t*_*2g*_ bands (<0.2 eV) are much smaller than the band gap (>1.2 eV). These results imply that *α*-RuCl_3_ is not a quasimolecular-orbital insulator; otherwise, the *t*_*2g*_ band distances and the band gap would exhibit a common energy scale of *d*-*d* hopping^21^. For comparison, we overlaid the band dispersions based on the results of LDA + SOC + *U* calculations; the result is shown in the right-hand side of Fig. 1(d). The red and blue solid lines correspond to the Ru *t*_*2g*_ bands and the Cl 3*p* bands, respectively. The calculations also support our finding that the flat Ru 4*d* and the dispersive Cl 3*p* bands are located near and much below the *E*_F_, respectively. Despite this success, some discrepancies between the ARPES spectra and the calculation results exist, as discussed below.

**Figure 2:**
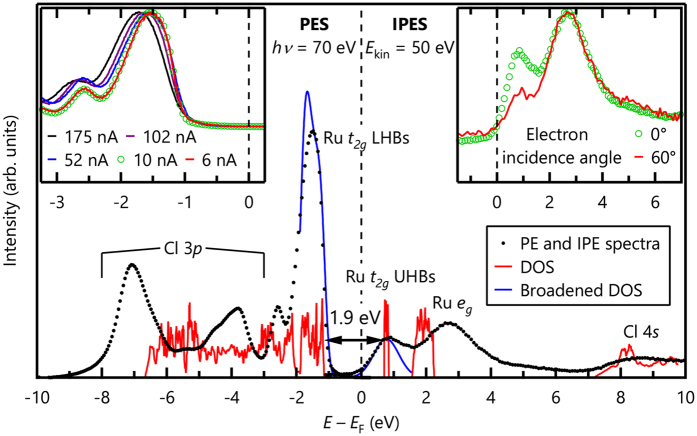
PE and IPE spectra of *α*-RuCl_3_. The red and blue solid lines represent the density of states and the broadened one from LDA + SOC + *U* calculations, respectively. By comparing the experimental and theoretical results, we estimated the size of the band gap to be approximately 1.9 eV. Note that the crystal field splitting is underestimated in the LDA + SOC + *U* calculations. The top-left and top-right insets show source-flux dependence in PE and IPE spectra, respectively. The PE and IPE spectra are measured at the conditions (10 nA and electron incidence angle = 0°) under which charging effects are negligible.

To resolve the controversy regarding the size of the band gap, we used a combination of angle-integrated PE and IPE spectroscopies. Note that the PE and IPE spectra contain information about the density of states of the occupied and unoccupied bands, respectively. Therefore, the combination of PE and IPE spectroscopies has been established as the most direct method for determining an electronic energy gap^23^. To avoid possible sample charging effects, we adjusted source fluxes for both ARPES and IPE experiments as shown in the insets of Fig. 2 (see the Method section for a detailed description). The black dots in Fig. 2 show both the PE and IPE spectra of *α*-RuCl_3_. The PE spectrum was obtained from the ARPES data of Fig. 1(d) by integrating over the momentum. On the basis of the arguments for Fig. 1(d), we assign the peak at approximately −1.5 eV to the Ru *t*_*2g*_ antibonding lower Hubbard bands (LHBs). The two strong peaks at approximately −4.0 and −7.0 eV should originate from the Cl 3*p* nonbonding and bonding bands, respectively. The right-hand side of Fig. 2 shows an IPE spectrum. Two prominent peaks near the *E*_F_ are assigned to the Ru *t*_*2g*_ upper Hubbard bands (UHBs) and Ru *e*_*g*_ bands. The crystal-field splitting 10*Dq*, which is the energy separation between the Ru *t*_*2g*_ UHBs and *e*_*g*_ bands, is estimated to be approximately 2.2 eV. This value is similar to that observed in an x-ray absorption spectrum^16^.

We estimate the band gap of *α*-RuCl_3_ to be 1.9 eV, which is much larger than the values reported in earlier studies^15^,^16^,^17^,^18^. In principle, the size of the band gap should correspond to the energy range of zero intensity in PE and IPE spectra. However, the range of zero intensity is reduced because of hole or electron lifetime and experimental spectral broadening. Usually, the position of a band edge is experimentally determined by the half maximum or by the intersection point of a linear extrapolation of the leading edge. The size of the errors for those methods is approximately a half of the experimental resolution. If the resolution is not good enough as in the IPE spectrum, the error will be comparable to the band-gap size. To reduce the error, we utilized the density of states from the LDA + SOC + *U* calculations, which was broadened by experimental resolutions as shown in blue lines of Fig. 2. To reproduce the PE and IPE spectra near the *E*_F_, we used *U* − *J*_H_ = 4.5 eV in the calculations. (*J*_H_ is the Hund’s coupling). As shown in Fig. 2, the LDA + SOC + *U* can explain both the valence and conduction bands near the *E*_F_ reasonably well. On the basis of this comparison, we assigned the valence band maximum and the conduction band minimum. Then, the band gap of *α*-RuCl_3_ should be approximately 1.9 eV. Note that this magnitude is clearly much higher than the 0.3 eV value obtained via a Hall-effect study^15^, and also higher than the 1.0 eV value from recent optical studies^17^. The former may be related with an activation gap due to defect states, and the latter implies strong exciton effect that is observed in alkali halides^24^ and other transition-metal compounds^25^.

**Figure 3:**
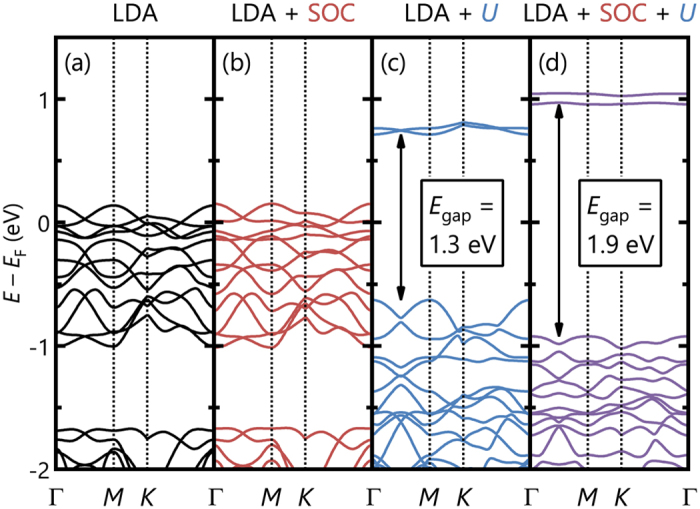
Electronic band structures of *α*-RuCl_3_ by changing *U* and SOC. (**a**) LDA, (**b**) LDA + SOC, (**c**) LDA + *U*, and (**d**) LDA + SOC + *U* calculations. Note that the band-gap value of approximately 1.9 eV can be explained only when both *U* and SOC terms are included.

We found that the interplay between the Coulomb interaction *U* and SOC is essential for understanding the physics of *α*-RuCl_3_. To clarify the roles of these effects, we performed LDA calculations with and without the *U* and SOC terms. Figures 3(a)–(d) display the results of LDA, LDA + SOC, LDA + *U*, and LDA + SOC + *U* calculations. As shown in Fig. 3(a), in the absence of *U* and SOC, the partially filled Ru bands with *t*_2g_^5^ electrons should result in a metallic ground state. As shown in Fig. 3(b), the system still remains metallic when SOC is included in the calculations. Nevertheless, the narrow *t*_*2g*_ bands repel each other because of SOC, resulting in an apparent total *t*_*2g*_ bandwidth broadening, as reported previously^26^. By contrast, as shown in Fig. 3(c), the LDA + *U* results predict a gapped electronic structure, indicating the prime importance of the Coulomb interaction for the insulating nature of *α*-RuCl_3_. However, the predicted gap size is only approximately 1.3 eV. We can properly describe the observed energy gap value of 1.9 eV only when we include both SOC and *U*. The large enhancement of the gap size by 0.6 eV due merely to the introduction of a small SOC of 0.13 eV indicates that SOC plays a crucial role in the electronic structure of *α*-RuCl_3_, especially near the Fermi level^27^.

Although the PE and IPE spectra can be explained reasonably well by the LDA + SOC + *U* calculation results, some discrepancies still exist. In Figs 1(d) and 2, a sharp nondispersive peak that cannot be explained by the calculations is observed at approximately −2.5 eV. The orbital character of this peak appears to be Ru 4*d* because its intensity change with varied photon energies is similar to those of the main Ru *t*_*2g*_ bands^22^. Moreover, the clear separation between the Ru 4*d* and the Cl 3*p* bands in the ARPES spectrum cannot be reproduced in the calculations. As shown in the IPE spectrum of Fig. 2, the size of the crystal-field splitting is also underestimated in the LDA + SOC + *U* results.

To gain further insights, we carried out CI calculations for a single RuCl_6_^3−^ cluster, in which we considered the Ru 4*d* and the Cl 3*p* bonding orbitals while taking the full multiplet structures into account. Whereas a local cluster calculation study of the *α*-RuCl_3_ has been reported in the literature, this work only considered the *d*^5^ ground state multiplets that are not directly related to the PE and IPE spectra^28^. To reduce the dimension of the Hilbert space, the Cl nonbonding states at approximately −4 eV are not considered in our calculations. The relevant Hamiltonian has numerous parameters, including *U, J*_H_, *λ*_SOC_, 10*Dq*, charge-transfer energy Δ for the excitation from the Cl 3*p* to Ru *t*_*2g*_ orbitals, and Slater-Koster parameters *t*_*pdσ*_ and *t*_*pdπ*_. However, many of these parameters can be determined unambiguously, i.e., the *λ*_SOC_ value was adopted from an inelastic neutron scattering study^10^, the 10*Dq* value from the energy difference between the Ru *t*_*2g*_ UHBs and the Ru *e*_*g*_ peak, and the Δ value from the energy difference between the Cl nonbonding states and the Ru *t*_*2g*_ UHBs. In most transition-metal compounds, *t*_*pdσ*_ is approximately two times larger than *t*_*pdπ*_^29^. Then, the values of the remaining three parameters, *U, J*_H_, and *t*_*pdπ*_, can be obtained with minor errors by fitting the band gap, the −2.5 eV peak position, and the position of the 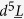 final states at approximately −7 eV. The obtained parameter values are listed in Table 1. Note that the magnitudes of *U, J*_H_, and Δ are difficult to determine without spectroscopic data because of dynamical screening^30^,^31^.

**Figure 4:**
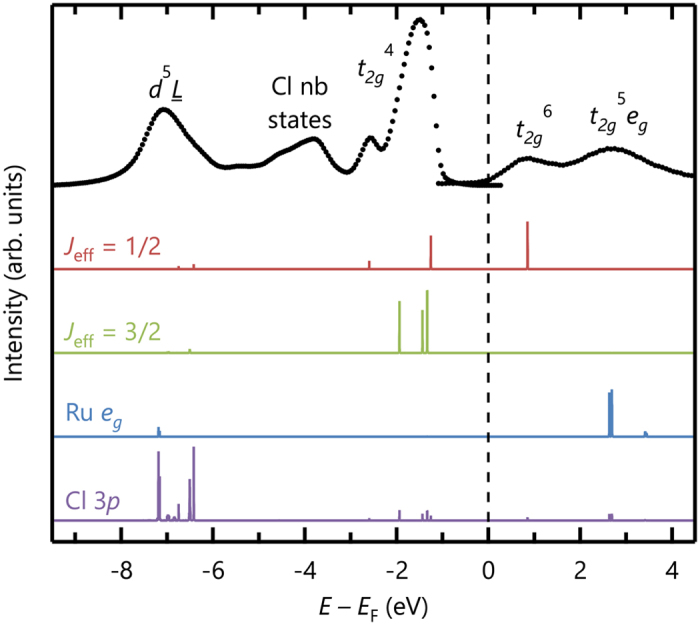
Comparison of PE and IPE spectra obtained experimentally and from CI calculations for (**a)** RuCl_6_^3−^ cluster. Spectral weights from CI calculations are shown separately by their spin-orbital characters in the ground state. The electronic energy gap is determined solely by excited states from the *J*_eff_ = 1/2 state. Note that nonbonding Cl 3*p* orbitals are not included in the calculations; thus, no peak is observed at approximately −4 eV.

Our CI calculations can explain the spectral features of the PE and IPE spectra that were difficult to explain on the basis of LDA + SOC + *U* calculations. As shown in Fig. 4, the positions of the energy levels determined from the CI calculations are in good agreement with the peak positions in the PE and IPE spectra. Despite the moderate SOC, the electronic structure of *α*-RuCl_3_ is governed by *J*_eff_ = 1/2 physics because the electronic energy gap is determined by excited hole and electron states that solely originate from the *J*_eff_ = 1/2 state. Among five multiplets of *t*_*2g*_^4^ configurations, curious −2.5 eV peak not explained by the band calculations emerges as a *J*_eff_ = 1/2 state. The unusual high-binding energy of *J*_eff_ = 1/2 state originates from the strong Hund’s coupling of Ru *d* orbitals (refer to the Supplemental Material of ref. 19 for a detailed description). The agreement between the *J*_eff_ = 1/2 nature and a large *U* signifies that the 4*d* compound *α*-RuCl_3_ has a strong local nature and a relativistic Mott ground state instead of the quasimolecular-orbital insulating state^19^.

The values of physical parameters obtained from the CI calculations can be utilized to study the Kitaev physics in *α*-RuCl_3_. The strengths of the Heisenberg (*J*), Kitaev (*K*), and off-diagonal (Γ) exchange interactions in the Heisenberg-Kitaev model are easily obtained^2^,^3^,^32^ from our values for the physical parameters listed in Table 1. The single shortcoming of our approach is that our CI calculations were performed on a single-site RuCl_6_^3−^ cluster and therefore did not include the direct *d-d* hopping terms between the nearest-neighbor Ru ions. To obtain the exchange interaction terms, we adopted the values of the *d*-*d* hopping parameters *t*_*ddσ*_ and *t*_*ddπ*_ from a recent theoretical study^33^. The exchange strengths of *J, K*, and Γ are then determined to be −0.7, −1.6, and 1.5 meV, respectively. The magnitudes of these values are much smaller than those estimated from inelastic neutron scattering experiments^10^ but are similar to those obtained from recent quantum chemistry calculations based on an assumed *P*3_1_12 structure^28^. To be more precise, performing the CI calculations with a full set of Ru 4*d* orbitals for a multi-site cluster is highly desirable^19^; however, this approach requires a much larger Hilbert space.

## Conclusion

In conclusion, we investigated the electronic structure of the Kitaev candidate material *α*-RuCl_3_. By combining both photoemission and inverse photoemission studies, we directly measured a band gap of 1.9 eV in *α*-RuCl_3_; this band gap is much larger than values reported previously. We also showed that the interplay between the electron correlation and SOC plays a crucial role in determining the nature of the Mott insulating ground state of *α*-RuCl_3_. By taking into account the many-body effects using CI calculations for a RuCl_6_^3−^ cluster, we obtained the physical parameters and exchange-interaction strengths of the Heisenberg-Kitaev model. The obtained parameters will provide a useful guide for the synthesis of Kitaev materials with the quantum-spin-liquid state. For example, the application of pressure or strain could be a strategy for achieving this goal. The evolution of the system due to the perturbations on the Kitaev phase diagram strongly depends on details of the parameters obtained in this study.

## Methods

### Experiments

Single-crystalline samples of *α*-RuCl_3_ were grown by the self-chemical vapor transport method. Their crystallinity was confirmed by Laue diffraction. All samples were cleaved *in situ* for ARPES and IPE measurements. ARPES measurements were performed at the Beamline 4A1 of Pohang Light Source. ARPES spectra were obtained at the photon energy of *hν* = 70 eV and with a total energy resolution of 50 meV. During the measurements, the sample temperature was maintained at 280 K under a vacuum of 3 × 10^−11^ Torr. IPE measurements were carried out at HiSOR^34^,^35^. The incident electron kinetic energy was set to 50 eV and an energy resolution was 0.9 eV. The sample temperature was 340 K under a vacuum of 3 × 10^−10^ Torr. IPE spectra were obtained in the normal incidence mode. The angular divergence of the electron beam was approximately 4°, corresponding to approximately one-third the length of the Γ*K* line. The Fermi levels and total experimental resolutions of ARPES and IPE spectra were determined by measuring the Fermi edge of Au electrically contacted to the sample. We checked sample charging effects in both PE and IPE spectra by varying source fluxes. The results are shown in the insets of Fig. 2. In PE spectra, when the photon flux is smaller than 10 nA, the spectral shift and the line-shape change due to charging becomes negligible. In IPE spectra, we changed the electron flux per unit area by changing the electron incident angle. As shown in the top-right inset of Fig. 2, the shift of the IPE spectrum was negligible even when the incidence electron density on the sample surface is decreased by approximately two times. The PE and IPE spectra in the main text were taken at 10 nA and the electron incidence angle of 0°, respectively.

### Theory

To calculate the band structure, we used the density functional theory code OPENMX (http://www.openmx-square.org) with a zigzag magnetic ordering that was reported to occur in *α*-RuCl_3_^11^,^36^. In the LDA + *U* and LDA + SOC + *U* calculations, the *U* − *J*_H_ value of 4.5 eV was used to reproduce the PE and IPE spectra near the *E*_F_. To explain the fine details of the PE and IPE spectra, we also performed CI calculations^37^ for a local RuCl_6_^3−^ cluster while neglecting the nonbonding Cl 3*p* molecular orbitals. We solved the Hamiltonian for a five-hole system using the Lanczos exact diagonalization method and calculated the one-particle Green’s functions by spanning the eigenvalues of four- and six-hole systems using the band Lanczos method^38^.

## Additional Information

**How to cite this article**: Sinn, S. *et al*. Electronic Structure of the Kitaev Material *α*-RuCl_3_ Probed by Photoemission and Inverse Photoemission Spectroscopies. *Sci. Rep.* **6**, 39544; doi: 10.1038/srep39544 (2016).

**Publisher's note:** Springer Nature remains neutral with regard to jurisdictional claims in published maps and institutional affiliations.

## Figures and Tables

**Table 1:** Physical parameters of CI calculations.

*U*	*J*_*H*_	*λ*_SOC_	10*Dq*	Δ	*t*_*pdσ*_	*t*_*pdπ*_
4.35	0.35	0.13	2.2	5.0	1.90	−0.90

Units are in eV. The parameters were determined by reproducing the experimental PE and IPE spectra in Fig. 2 with CI calculations.
